# Phenotypic and Genotypic Characterization of Clinical Isolates of Carbapenem-Resistant Acinetobacter baumannii: A Cross-Sectional Study From a Tertiary Care Hospital in Western Uttar Pradesh, India

**DOI:** 10.7759/cureus.83998

**Published:** 2025-05-12

**Authors:** Prachi Gupta, Amit Singh, Anuj Gupta, Dharmendra Prasad Singh, Sulekha Nautiyal

**Affiliations:** 1 Microbiology, Graphic Era Institute of Medical Sciences, Dehradun, IND; 2 Microbiology, Uttar Pradesh University of Medical Sciences, Etawah, IND; 3 Forensic Medicine, Graphic Era Institute of Medical Sciences, Dehradun, IND; 4 Microbiology, Shri Guru Ram Rai Institute of Medical and Health Sciences, Dehradun, IND

**Keywords:** blaimp, blambl, blandm, blaoxa, blaoxa-23, blaoxa-51, blavim, carbapenem-resistant acinetobacter baumannii

## Abstract

Introduction

*Acinetobacter baumannii,* due to its recognition as a major cause of healthcare-associated infections, is posing a huge therapeutic challenge for clinicians globally. The present study aimed to study the phenotypic and genotypic characteristics of carbapenem-resistant *A. baumannii *(CRAB).

Methods

A total of 121 clinical isolates of *A. baumannii *were identified using VITEK 2 (bioMérieux, France) from January 2017 to June 2018. Antimicrobial susceptibility testing was performed by modified Kirby-Bauer disc diffusion. CRAB isolates were subjected to modified Hodge test (MHT), combined disk test (CDT), and double disk synergy test (DDST) for the detection of carbapenemase production, followed by determination of minimum inhibitory concentration (MIC) of meropenem and imipenem by agar dilution and E-test, respectively. Molecular characterisation was done by polymerase chain reaction (PCR) for the detection of blaOXA (blaOXA-51, blaOXA-23, blaOXA-24, and blaOXA-58) and blaMBL (blaVIM, blaIMP, blaNDM, blaSIM, blaSPM, and blaGIM) genes.

Results

The study showed that 34.71% of *A. baumannii* isolates were CRAB. On MHT, 64.29% isolates were identified as carbapenemase producers. DDST and CDT show 40.48% of CRAB isolates as metallo-β-lactamase (MBL) producers, and one isolate was identified as an MBL producer by DDST only. MIC_50 _and MIC_90_ values of meropenem were 16 and 32µg/mL, respectively. MIC_50 _and MIC_90_ values of imipenem were 12 and >32µg/mL, respectively. blaOXA-51 was detected in all the isolates. blaOXA-23 was detected in 73.81% and blaNDM in 21.43% of isolates. blaVIM, blaOXA-58, blaSIM, blaIMP, and blaSPM were also detected in 7.14%, 4.76%, 2.38%, 2.38%, and 2.38% isolates, respectively. Neither blaOXA-24 nor blaGIM was detected in any of the study isolates.

Conclusion

A high level of carbapenem resistance was found in *A. baumannii.* blaOXA-23 was the most common carbapenamase gene, followed by blaNDM.

## Introduction

*Acinetobacter* species, particularly *A. baumannii,* have emerged as a significant nosocomial pathogen causing a multitude of infections that include ventilator-associated pneumonia, wound infections, bloodstream infection, urinary tract infections, surgical-site infections, and meningitis [1].

*A. baumannii* is inherently resistant to several antibiotics like ampicillin, narrow-spectrum cephalosporins, trimethoprim, and ertapenem [2]. *Acinetobacter* spp. have a magnificent capacity to develop multiple resistance mechanisms against major antibiotic classes - they have become resistant to broad-spectrum β-lactams; third-generation cephalosporins, carboxypenicillins, aminoglycosides, fluoroquinolones, and increasingly to carbapenems [2].

The rising incidence of multidrug resistance in this pathogen is therapeutically threatening hospitalized patients by restricting the availability of antimicrobial options. Carbapenems have long been considered antimicrobials of last resort for the treatment of infections caused by *A. baumannii*. However, in recent years, owing to the selective pressure created by extensive use of carbapenems, the number of isolates exhibiting resistance to carbapenems has expanded worldwide, leaving us with restricted treatment choices (i.e., tigecycline and colistin) [2]. Recently published studies have reported a carbapenem-resistant rate of *A. baumannii* as 40-75% throughout India [3].

The carbapenem resistance in *A. baumannii* is predominantly mediated by production of carbapenemases, particularly OXA-type carbapenem-hydrolyzing β-lactamases (class D of Ambler classification) and metallo-β-lactamases (MBLs) (class B of Ambler classification). Four families of OXA genes have been recognised in *A. baumannii*: blaOXA-23-like (blaOXA-23, blaOXA-27, and blaOXA-49); blaOXA-24-like (blaOXA-24, blaOXA-25, blaOXA-26, and blaOXA-40); blaOXA-58-like; and blaOXA-51-like [1]. The blaOXA-51 is intrinsic to *A. baumannii* and is chromosomally encoded, hence used as a genotypic marker for the identification of this species [2,4]. Among MBLs, four types are known in *A. baumannii* that include New Delhi metallo-β-lactamase (NDM), imipenemase (IMP), Seoul imipenemase (SIM), and Verona integron-encoded metallo-β-lactamase (VIM) [1]. Recently published studies from India have reported the presence of blaGIM in *A. baumannii* [5-7].

In the present study, the clinical isolates of carbapenem-resistant *A. baumannii* (CRAB) from a tertiary-level healthcare institute in western Uttar Pradesh were studied for the presence of OXA-type β-lactamases (blaOXA­-23, blaOXA­-24, blaOXA-51, and blaOXA-­58) and blaVIM, blaIMP, blaNDM, blaSPM, blaSIM, and blaGIM MBL genes.

## Materials and methods

Study design and site

We conducted a hospital-based cross-sectional study over a period of 18 months (January 2017 to June 2018) in the Department of Microbiology at Uttar Pradesh University of Medical Sciences (UPUMS), Etawah, India, after obtaining the ethical approval from institute's ethical committee vide letter No. 260/UPUMS/Dean/2017-18/2017/147 dated 26 July 2017.

Identification of *Acinetobacter* species

In the present study, 121 non-repetitive *A. baumannii* were isolated and identified by VITEK 2 (bioMérieux, France) from various clinical specimens received routinely for culture and sensitivity in the microbiology laboratory from indoor and outdoor patients during the above-mentioned period.

Antimicrobial susceptibility testing (AST)

AST was performed by the modified Kirby-Bauer disc diffusion method according to the Clinical and Laboratory Standards Institute (CLSI) 2017 guidelines [8]. Commercially available antibiotic disks of ampicillin (10µg), gentamicin (10µg), ciprofloxacin (5µg), piperacillin (100µg), cefexime (5µg), cefepime (30µg), ceftriaxone (30µg), aztreonam (30µg), imipenem (10µg), meropenem (10µg), tigecycline (15µg), cefoperazone/salbactum (75/30µg), piperacillin/tazobactum (100/10µg), polymyxin B (300 units), colistin (10µg), and nitrofurantoin (50µg, in urinary isolates) were used. CLSI 2017 guidelines [8] were used to interpret the zone of inhibition and classify the organism as susceptible, intermediate susceptible, or resistant. Isolates resistant to imipenem and/or meropenem were identified as CRAB. These were subjected to subsequent testing. However, CLSI guidelines are unavailable for colistin, polymyxin B, and tigecycline against *Acinetobacter*. Keeping the breakpoints of ≤2 as sensitive and ≥4 as resistant, the zone sizes of colistin and polymyxin B in the disc diffusion test were taken as ≥11mm as susceptible and ≤10mm as resistant [5]. The interpretation for tigecycline was ≥16mm was considered sensitive, and ≤12mm was considered resistant [9].

Modified Hodge test (MHT)

CRAB isolates were subjected to MHT for the detection of carbapenemases according to CLSI 2017 guidelines [8]. The presence of indentation in the growth of the indicator strain towards the imipenem disk on either side of the test isolate was interpreted as a positive result.

Combined disk test (CDT) imipenem-ethylenediaminetetraacetic acid (EDTA)

The imipenem-EDTA CDT was performed as described by Yong et al. [10]. If the increase in inhibition zone with the imipenem and EDTA disk was ≥7mm than the imipenem disk alone, the isolate was considered an MBL producer.

Double disk synergy test (DDST) imipenem-EDTA

The imipenem-EDTA DDST was performed according to a study by Lee et al. [11]. Enhanced zone of inhibition in the region between imipenem and EDTA disks, as compared to the zone of inhibition on the other side of the imipenem disk, indicated a positive result.

Minimum inhibitory concentration (MIC) of meropenem by agar dilution method

Agar dilution was used to determine the meropenem MIC of CRAB isolates. Mueller-Hinton agar (MHA) plates containing meropenem in twofold dilutions from 0.25µg/mL to 64µg/mL were used. For quality control strains, *Escherichia coli* ATCC 25922 (meropenem susceptible) and *Pseudomonas aeruginosa* ATCC 27853 (meropenem resistant) were used. Isolates with MICs of ≥8µg/mL were interpreted as resistant to meropenem, while those with MICs ≤2µg/mL were considered susceptible, and those with values between the two cut-offs were regarded as intermediate susceptible according to the CLSI 2017 guidelines [8].

MIC of imipenem by E-test

The commercially available imipenem E-test strip containing a dilution range of imipenem from 0.002 to 32µg/mL was used, according to the manufacturer’s instructions. MIC was interpreted according to the CLSI 2017 guidelines [8]. MIC value of <2µg/mL was considered as susceptible, 4µg/mL was considered as intermediate susceptible, and ≥8µg/mL was interpreted as resistant. Following the manufacturer's instructions, when the MIC reading fell between two consecutive twofold dilutions, it was rounded up to the next higher twofold dilution.

Molecular characterization of *A. baumannii* with reference to carbapenemases

Genomic DNA was purified from the study isolates by the simple boiling method [12]. The purified DNA was stored at -20 degrees Celsius till further processing. Sequences of primers used for the detection of OXA carbapenemase [13]; metalo-beta-lactamases like blaVIM, blaIMP, blaSPM, blaSIM, blaGIM [14]; and blaNDM [15] are given in Table 1.

**Table 1:** PCR primers used for the detection of OXA carbapenemase and metallo-beta-lactamases PCR: polymerase chain reaction

Primer	Gene	Primer sequence (5′-3′)	Product size (bp)
blaOXA-23 like - F	blaOXA-23 like	GAT CGG ATT GGA GAA CCA GA	501
blaOXA-23 like - R		ATT TCT GAC CGC ATT TCC AT	
blaOXA​​​​​​​-51 like - F	blaOXA-51 like	TAA TGC TTT GAT CGG CCT TG	353
blaOXA​​​​​​​-51 like - R		TGG ATT GCA CTT CAT CTT GG	
blaOXA​​​​​​​-24 like - F	blaOXA-24 like	GGT TAG TTG GCC CCC TTA AA	246
blaOXA​​​​​​​-24 like - R		AGT TGA GCG AAA AGG GGA TT	
blaOXA​​​​​​​-58 like - F	blaOXA-58 like	AAG TAT TGG GGC TTG TGC TG	599
blaOXA​​​​​​​-58 like - R		CCC CTC TGC GCT CTA CAT AC	
blaVIM-F	blaVIM	GAT GGT GTT TGG TCG CAT A	390
blaVIM-R		CGA ATG CGC AGC ACC AG	
blaIMP-F	blaIMP	GGA ATA GAG TGG CTT AAT TCT C	188
blaIMP-R		CCA AAC CAC TAC GTT ATC T	
blaSPM-F	blaSPM	AAA ATC TGG GTA CGC AAA CG	271
blaSPM-R		ACA TTA TCC GCT GGA ACA GG	
blaGIM-F	blaGIM	TCG ACA CAC CTT GGT CTG AA	477
blaGIM-R		AAC TTC CAA CTT TGC CAT GC	
blaSIM-F	blaSIM	TAC AAG GAA TTC GGC ATC G	570
blaSIM-R		TAA TGG CCT GTT CCC ATG TG	
blaNDM-F	blaNDM	ACC GCC TGG ACC GAT GAC CA	264
blaNDM-R		GCC AAA GTT GGG CGC GGT TG	

Multiplex polymerase chain reaction (PCR) for blaOXA genes

Multiplex conventional PCR was performed for the detection of the four families of OXA-type (blaOXA-23-like, blaOXA-24-like, blaOXA-51-like, and blaOXA-58-like) carbapenamases found in *A. baumannii*. The 50µL PCR reaction mixture contained 5µL of 10X PCR buffer, 4µL of 10mM dNTPs (2.5mM each), 1µL of 25mM MgCl2, 0.5µL of each of the forward and reverse primers, 0.5µL of 1.5U Taq DNA polymerase, and 1µL of test DNA template. PCR protocol followed was 94°C for five minutes (initial denaturation), 33 cycles of 94°C for 25s, 53°C for 40s, and 72°C for 50s, followed by 72°C for six minutes (final elongation). The PCR products of 501bp (blaOXA-23-like), 353bp (blaOXA-51-like), 246bp (blaOXA-24-like), and 599bp (blaOXA-58-like) were visualized by 2% agarose gel electrophoresis containing 0.5μg/mL ethidium bromide. A 100bp DNA ladder was used for comparison [13].

Multiplex PCR for blaMBL genes (blaVIM, blaIMP, blaSIM, blaSPM, and blaGIM)

Second multiplex conventional PCR was done for the detection of MBL genes (blaVIM, blaIMP, blaSIM, blaSPM, and blaGIM). The final 50µL PCR mixture contained 5µL 10X PCR buffer, 4µL of 10mM dNTPs (2.5mM each), 1µL of 25mM MgCl2, 0.5µL of each of the forward and reverse primers, 0.5µL of 1.5U Taq DNA polymerase, and 1µL of DNA template of the test strain. Thermal cycling was programmed at 94°C for five minutes (initial denaturation), 36 cycles of 94°C for 30s, 52°C for 40s, and 72°C for 50s, followed by 72°C for five minutes (final elongation step). The amplified products of 188bp (blaIMP), 390bp (blaVIM), 570bp (blaSIM), 271bp (blaSPM), and 477bp (blaGIM) were visualized by 2% agarose gel electrophoresis containing 0.5μg/mL ethidium bromide. A 100bp DNA ladder was used as a molecular weight marker [14].

PCR for blaNDM MBL gene

Single conventional PCR was performed for the detection of the blaNDM MBL gene. The final 50μL PCR mixture contained 10μL of 10x PCR buffer, 10μL of 2mM dNTPs, 5μL of 2.5 mM MgCl2, 1μL each of the forward and reverse primers, 2μL of 1U Taq DNA polymerase, and 2μL of genomic DNA of the test strain. PCR conditions used were 94°C for five minutes (initial denaturation), 35 cycles of 95°C for 30s, 58°C for 30s, 72°C for 30s, 72°C for 10 min (final extension). The PCR products of 264bp (blaNDM) were visualized by 2% agarose gel electrophoresis containing 0.5μg/mL ethidium bromide and compared with 100bp DNA ladder [15].

## Results

Of the 121 isolates of *A. baumannii* isolated during the study period, 42 (34.71%) were found to be resistant to both imipenem and meropenem and identified as CRAB. None of the isolates was found to be resistant to only one of these drugs. All these 42 isolates were derived from indoor patients, of which 57.14% (24/42) were admitted to the ICU. Of these 42 CRAB isolates, 38.09% (16/42) were from respiratory samples (endotracheal (ET) aspirates, bronchoalveolar lavage (BAL) fluid, and sputum). CRAB isolates were found more commonly from male patients as compared to female patients (M:F=1.47:1) and from young adults belonging to the age group 21-30 years (22/42, 52.38%).

Antimicrobial susceptibility testing showed a high level of resistance against the antibiotics tested. All the strains were found to be resistant to ampicillin, cefexime, ceftriaxone, cefoperazone-salbactum, and piperacillin. All urinary isolates showed resistance to nitrofurantoin. All isolates showed susceptibility to tigecycline, polymyxin B, and colistin. Variable resistance was observed for gentamicin (80.95%), ciprofloxacin (88.06%), cefepime (90.47%), piperacillin-tazobactum (92.86%), aztreonam (90.47%).

On MHT, 64.29% (27/42) of CRAB isolates were identified as carbapenemase producers. Of all the study isolates, 40.48% (17/42) were identified as MBL producers by both DDST and CDT, and 2.38% (1/42) by DDST only. The MICs of meropenem for CRAB isolates were found to range from 4 to >64µg/mL. The majority of these isolates (40.48%, 17/42) had having MIC meropenem of 32µg/mL. MIC_50_ and MIC_90_ of meropenem were 16 and 32µg/mL, respectively. The MICs of imipenem for CRAB isolates ranged from 4 to >32µg/mL. The majority of these isolates (35.71%, 15/42) had having MIC imipenem of 12µg/mL. MIC_50_ and MIC_90_ of imipenem values were 12 and >32µg/mL, respectively.

**Figure 1:**
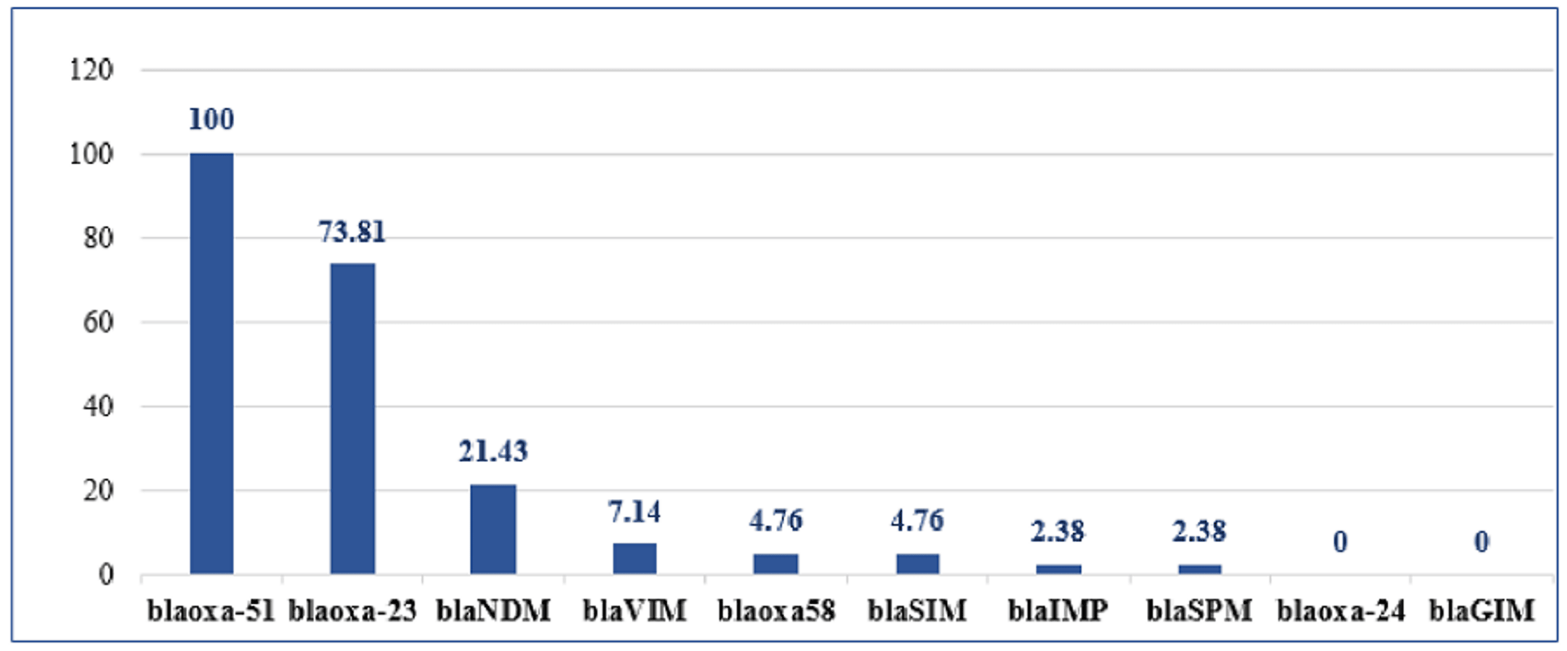
Distribution of various genes in CRAB isolates (in percentage) CRAB: carbapenem-resistant *Acinetobacter baumannii*

On PCR, all the CRAB isolates showed amplification for the blaOXA-51 gene, while none of the isolates showed amplification for blaOXA-24 and blaGIM. The distribution of various genes in them is shown in Figure 1.

**Figure 2:**
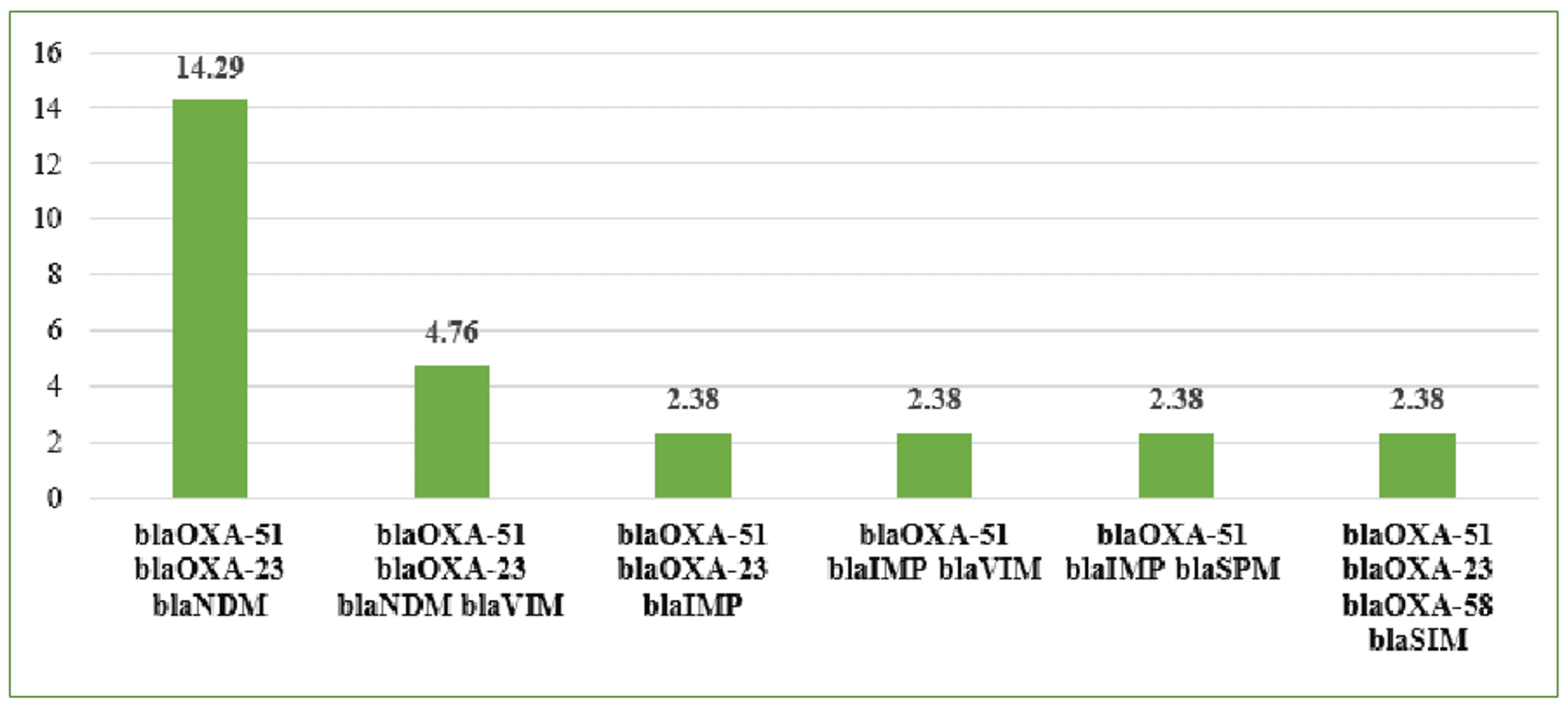
Distribution of various combinations of genes in CRAB isolates (in percentage) CRAB: carbapenem-resistant *Acinetobacter baumannii*

Different combinations of genes were also detected in CRAB isolates, most common being blaOXA-51, blaOXA-23, and blaNDM, as shown in Figure 2. It was also observed that 16.67% (7/42) isolates were positive only for blaOXA-51.

## Discussion

Multi-drug resistance in *A. baumannii* in healthcare-associated infections is a global concern. For a long time, carbapenems have been considered the preferred antimicrobial choice to combat infections caused by these multidrug-resistant (MDR) organisms.

Due to the extensive use of carbapenems to treat infections caused by MDR *A. baumannii*, there is an emergence of carbapenem resistance in *A. baumannii*. In the present study, 48.76% and 34.71% isolates of *A. baumannii* were found to be MDR and resistant to carbapenem, respectively. Recently published studies have reported the carbapenem-resistant rate of *A. baumannii* as 40-75% in various regions of India, such as Jammu and Kashmir, Delhi, Punjab, Maharashtra, and Tamil Nadu [3]. All the study isolates showed susceptibility to tigecycline, polymyxin B, and colistin. Similarly, 100% susceptibility to these drugs was also reported by a study done by Mohammed and Singh (2019) [7]. However, 97% and 93% susceptibility to polymyxin B and tigecycline, respectively, were reported by a study done by Amudhan et al. (2011) [16], indicating that these drugs should be used cautiously and reserved to treat infections in patients in whom all other antibiotics are showing resistance.

On MHT, 64.29% of CRAB isolates tested positive for carbapenemase production. Out of all the CRAB isolates, 42.86% and 40.48% tested positive for MBL production by DDST and CDT, respectively. The CLSI 2017 guidelines recommended MHT for the detection of carbapenemases only in members of the Enterobacteriaceae family but not for non-fermenting Gram-negative bacilli [8]. Several authors have found MHT with imipenem, EDTA, and ZnSO_4_ as a useful screening tool for carbapenemase production in *Acinetobacter* species [16,17]. However, the subsequent CLSI guidelines no longer recommend MHT as a phenotypic test for the detection of carbapenemases, even for members of the Enterobacteriaceae family. The CLSI 2024 guidelines recommend CarbaNP, modified carbapenem inactivation method (mCIM), and mCIM with EDTA-modified carbapenem inactivation method (eCIM) as phenotypic tests for the detection of carbapenemase in Enterobacterales and *P. aeruginosa* [18].

In the present study, CRAB isolates were found to have high MIC values for imipenem and meropenem. MIC_50_ and MIC_90_ values were 16 and 32µg/mL for meropenem and 12 and >32µg/mL for imipenem, respectively. In a study [16], Amudhan et al. reported the MIC_50_ and MIC_90_ values as 16 and 32µg/mL for meropenem and 32 and 64µg/mL for imipenem, respectively. Discrepancy was found between susceptibility to meropenem by disc diffusion and agar-dilution, 4.76% of the isolates resistant to meropenem on disc diffusion test had MICs in the intermediate range, while 95.24% had MICs in the resistant range. Again, discrepancy has been noted between susceptibility to imipenem by disc diffusion and E-test; 14.29% of the isolates resistant to imipenem on disc diffusion test had MICs in the intermediate range, while 85.71% had MICs in the resistant range. These findings suggest that E-test and agar dilution tests are more reliable than disc diffusion for testing of susceptibility of antibiotics.

blaOXA-51 has been identified as a genotypic marker for species identification of *A. baumannii* [2,4]. All the study isolates in this study showed the presence of blaOXA-51, supporting the use of this gene for speciation of *A. baumannii* by genotypic methods.

In the present study, blaOXA-23 (73.81%) was the predominant gene found in CRAB isolates. Many studies on molecular characterisation of enzyme-mediated resistance mechanisms of *A. baumannii* across India also reported similar findings [5,16,19-26]. After blaOXA-23, blaNDM (21.43%) was the most common gene found in this study, which is again in concordance with findings of various Indian studies done in the recent past [7,20,21,23-26]. While a study from Pondicherry [19] did not find blaNDM in their study isolates, which may be due to the fact that the study was done in 2009-2010, when blaNDM was not widely distributed in India. Another study from Chennai also did not report the presence of blaNDM in their study isolates [6].

In the present study, blaOXA-58 (4.76%) was also found in a small number of isolates as reported in various Indian studies [16,20,24], and there are studies [19,21,23,25,26] where blaOXA-58 was not detected, indicating that this gene is not widely spread in India. blaOXA-24 was not found in any of our study isolates, which is in concordance with findings of a few other studies [20,23], while some Indian studies [16,19,21,24-26] reported the presence of blaOXA-24, which indicates that this gene is also not widely distributed in India.

After blaNDM, blaVIM (7.14%) was the second most common MBL gene found in CRAB isolates of this study. In the present study, blaIMP (4.76%), blaSIM (4.76%), and blaSPM (2.38%) were also detected in a few isolates, while blaGIM was not detected in any of the study isolates. Some studies reported the predominance of blaVIM [5,6] and others blaIMP [19,22]. Many Indian studies did not report the presence of blaIMP, blaSIM, and blaSPM in *A. baumannii* [7,20,21,23-25]. Few studies have reported the presence of blaGIM in CRAB isolates [5-7].

The co-existence of blaOXA and blaMBLs has been reported from several geographic regions. In this study, different combinations of OXA and MBL genes were also found, the most common being blaOXA-23 with blaNDM, followed by blaOXA-23 with blaVIM. This finding is in concordance with a study [24] done by Vijayakumar et al., in which isolates of *A. baumannii* were collected from eight centres across India, and another study [26] from Cochin also reported blaOXA-23 and blaNDM as the most common combination.

In the present study, co-existence of MBL genes was also found, blaNDM with blaVIM, and blaIMP with blaSPM. In a study [16], Amudhan et al. found 42.24% of isolates carrying blaVIM with blaOXA-23 and 0.86% of isolates carrying blaVIM, blaIMP, and blaOXA-23 genes.

In the present study, 16.67% of the CRAB isolates showing PCR negative results for carbapenemase genes (other than blaOXA-51) may possess other enzymes mediating carbapenem resistance, like other less frequently encountered OXA genes or AmpC enzymes, and/or other mechanisms like altered membrane permeability and efflux mechanisms, which fall outside the scope of this study.

While this study was limited by being mono-centric, a small number of isolates, shorter duration, and PCR involving common carbapenem genes laid the initial foundation for the carbapenem resistance mechanism. An extensive study involving multiple centers, a large number of isolates, and over a longer duration will add more meaningful insights to the topic. Further incorporation of phenotypic tests like carbaNP, mCIM, eCIM, and genotypic assays targeting all the carbapenemase genes will give the complete picture.

## Conclusions

The study concludes that there is a high prevalence of CRAB among clinical isolates, predominantly from the ICU and respiratory samples, emphasizing the need for robust infection control practices. While the phenotypic tests gave an initial indication of carbapenemase and MBL production, molecular assays offered details about the underlying resistance mechanism. The blaOXA-23 gene is the predominant gene, followed by blaNDM, and their combination is the most frequently detected combination in CRAB. So the laboratories must be vigilant for detection of resistant isolates, especially CRAB, using phenotypic and molecular assays as described by the latest CLSI guidelines. Molecular detection of resistant genes helps us to track the spread of particular resistance genes within populations and the environment.
